# Remarkable Phenytoin Sensitivity in 4 Children with *SCN8A*-related Epilepsy: A Molecular Neuropharmacological Approach

**DOI:** 10.1007/s13311-015-0372-8

**Published:** 2015-08-09

**Authors:** Ragna S. Boerma, Kees P. Braun, Maarten P. H. van de Broek, Frederique M. C. van Berkestijn, Marielle E. Swinkels, Eveline O. Hagebeuk, Dick Lindhout, Marjan van Kempen, Maartje Boon, Joost Nicolai, Carolien G. de Kovel, Eva H. Brilstra, Bobby P. C. Koeleman

**Affiliations:** Department of Medical Genetics, University Medical Center Utrecht, Utrecht, The Netherlands; Department of Pediatric Neurology, University Medical Center Utrecht, Utrecht, The Netherlands; Department of Clinical Pharmacy, University Medical Center Utrecht, Utrecht, The Netherlands; Department of Neurology, University Medical Centre Groningen, Groningen, The Netherlands; Department of Pediatric Neurology, Stichting Epilepsie Instellingen Nederland, Zwolle, The Netherlands; Department of Neurology, Maastricht University Medical Center, Maastricht, The Netherlands; Epilepsy Center Kempenhaeghe, Heeze, The Netherlands

**Keywords:** *SCN8A*, phenytoin, epileptic encephalopathy, sodium channel blockers

## Abstract

Mutations in *SCN8A* are associated with epilepsy and intellectual disability. *SCN8A* encodes for sodium channel Nav1.6, which is located in the brain. Gain-of-function missense mutations in *SCN8A* are thought to lead to increased firing of excitatory neurons containing Nav1.6, and therefore to lead to increased seizure susceptibility. We hypothesized that sodium channel blockers could have a beneficial effect in patients with *SCN8A*-related epilepsy by blocking the overactive Nav1.6 and thereby counteracting the effect of the mutation. Herein, we describe 4 patients with a missense *SCN8A* mutation and epilepsy who all show a remarkably good response on high doses of phenytoin and loss of seizure control when phenytoin medication was reduced, while side effects were relatively mild. In 2 patients, repeated withdrawal of phenytoin led to the reoccurrence of seizures. Based on the findings in these patients and the underlying molecular mechanism we consider treatment with (high-dose) phenytoin as a possible treatment option in patients with difficult-to-control seizures due to an *SCN8A* mutation.

**Electronic supplementary material:**

The online version of this article (doi:10.1007/s13311-015-0372-8) contains supplementary material, which is available to authorized users.

## Introduction

Mutations in *SCN8A* have recently been described in patients with epileptic encephalopathy, intellectual disability, and developmental delay [1–4]. *SCN8A* encodes for the sodium channel Nav1.6, one of the major voltage-gated sodium channels (VGSC) in the central nervous system, playing an important role in action potential propagation and spread.

Other VGSC subtypes expressed in the human brain are Nav1.1, Nav1.2, and Nav1.3, encoded by *SCN1A*, *SCN2A*, and *SCN3A*, respectively. Nav1.3 primarily plays a role in the embryonic and early neonatal brain. Nav1.1 is located at inhibitory neurons, while Nav1.2 and Nav1.6 are the main channels in excitatory neurons [5]. Nav1.2 and Nav1.6 are located in the axon initial segment, which is where action potentials are initiated depending on the net result of excitatory and inhibitory signals the neuron receives. Nav1.6 regulates persistent and resurgent current and is voltage dependent. Persistent current is a steady state after firing of the cell, which is important for repetitive firing of the neuron, while resurgent current can occur after initial depolarization and prevents normal inactivation of the channel after depolarization, promoting the firing of another action potential. This allows neurons to fire quickly and repetitively [6]. These properties make Nav1.6 an important channel in neuronal excitability regulation.

In 2012, Veeramah et al. [1] were the first to describe a de novo *SCN8A* mutation in a patient with infantile epileptic encephalopathy. The patient had seizure onset at 6 months of age, developmental delay, features of autism, ataxia, and intellectual disability. She died at the age of 15 years from sudden unexplained death in epilepsy. Using whole genome sequencing, a p.Asn1768Asp mutation was detected in the patient, which was not found in her healthy parents nor her unaffected sibling. Functional studies showed persistent channel activation and incomplete channel inactivation, hereby contributing to increased firing [1]. After this publication, various other missense *SCN8A* mutations in patients with epileptic encephalopathy have been described [2, 4, 7, 8]. The only described *SNC8A* nonsense mutation resulting in haploinsufficiency was found in a patient with cerebellar atrophy, ataxia, and mental retardation, but without epilepsy [9]. Furthermore, functional studies of selected missense mutation detected in an epileptic encephalopathy patient both confirm gain-of-function and loss-of-function effects [3, 7, 8]. Given the fact that the Nav1.6 channel is mainly located in excitatory neurons [5], gain-of-function mutations would lead to increased firing of the neuron, making the brain more sensitive to epileptic discharges.

Dravet syndrome is caused by a loss-of-function mutation in *SCN1A*, which encodes for the Nav1.1 channel, located in inhibitory neurons. In these patients, a truncating mutation or deletion in *SCN1A* leads to a lack of well-functioning Nav1.1 channels. Given the fact that Nav1.1 channels are located in inhibitory neurons, this leads to impaired inhibition and therefore to increased excitation and epilepsy. Therefore, mutations in *SCN1A* and *SCN8A* both present clinically with epilepsy but probably have an opposite working mechanism (loss-of-function mutation leading to loss of inhibition, and gain-of-function mutation leading to increased excitability of excitatory neurons, respectively), both leading to increased excitability.

In patients with Dravet syndrome, sodium channel blockers are contraindicated, possibly because blockage of the remaining Nav1.1 will further impair inhibition and increase neuron firing. On the contrary, for patients with gain-of-function *SCN8A* mutations, we hypothesized that sodium channel blockers could be beneficial. Blocking the excessively firing Nav1.6 could decrease the excitability of the neuron and have a counteracting effect on the channel.

## Results

### Patient Descriptions and Response to Phenytoin

**Fig. 1:**
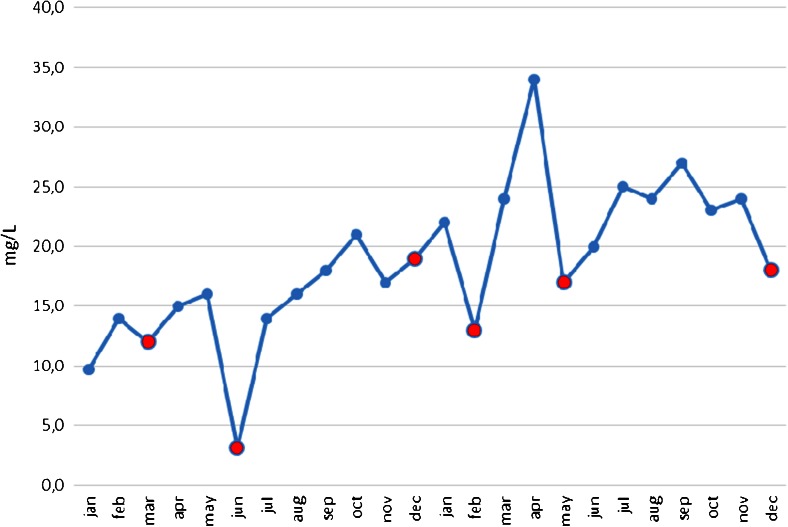
Phenytoin levels of patient 1. Red dots indicate hospital admissions for increased seizure frequency

We report on 4 patients with a de novo missense mutation in *SCN8A* and a remarkably good response on treatment with phenytoin and loss of seizure control when phenytoin medication was reduced (see Table 1 for patient characteristics). All patients had been antiepileptic drug resistant before the introduction of phenytoin. Three patients have been seizure-free since the initiation of phenytoin treatment, while seizure frequency and severity has decreased significantly in one patient (patient 1, Fig. 1). All mutations are reported using hg19 reference sequence and transcript ENST00000354534 and are predicted as damaging.

**Table 1:** Characteristics of 4 patients with an *SCN8A* mutation and phenytoin dependence

	Patient 1	Patient 2	Patient 3	Patient 4
Age	10 years	2 years	7 years	8 years
Age of onset	4.0 months	5.0 months	3.5 months	6.0 weeks
Type of seizures	Tonic with cyanosis; tonic–clonic	Tonic with cyanosis; atypical absence seizures	Tonic with apnea, bradycardia, cyanosis, status epilepticus	Tonic, tonic–clonic with apnea and cyanosis, status epilepticus, myoclonic
Seizure frequency before introduction of phenytoin	1–3 per day	1 per month to 3 per day	1 every 2 weeks to 5 per day, often clustered	10 per day
Previous AED	Valproate; carbamazepine; topiramate	Phenobarbital; valproate; levetiracetam; clobazam; topiramate	Valproic acid; clobazam; carbamazepine; oxcarbazepine	Pyridoxine, pyridoxal-phosphate, valproic acid, mysoline, clonazepam, midazolam
Current AED	Oxcarbazepine; phenytoin	Phenobarbital; valproate; phenytoin	Phenytoin	Phenobarbital; zonisamide; levetiracetam; clobazam; phenytoin
Phenytoin level at which seizure control achieved (mg/L)	Total > 30; free 2–3	Total 14–24; free 0.23–1.00	Total 12–20; free phenytoin level not determined	ND
Seizure-free on phenytoin	No, but drastically decreased seizure frequency	6 months	10 months*	5 years
Mutation	c.779T>C, p.Phe260Ser	c.4787C>G, p.Ser1596Cys	c.5610A>T, p.Glu1870Asp	c.2952C>G, p.(Asn984Lys)
MRI results	Normal	Normal	Cerebellar atrophy at age 7 years^†^	Loss of white matter volume
Psychomotor development	Moderate retardation	Delayed; no speech developed yet	Moderate retardation	Severe retardation, no speech, not able to sit
Other	Initially diagnosed with mitochondrial encephalopathy, because of decreased ATP production and decreased complex II and III activity; congenital hip dysplasia	Variant of unknown significance in KCNQ2, inherited from unaffected mother	Epiphysiolysis capitis femoris	Tethered cord, minor dysmorphisms, precocious puberty, scoliosis, severe feeding difficulties, kidney stones

AED = antiepileptic drug; ND = not determined; MRI = magnetic resonance imaging; ATP = adenosine triphosphate

*Recurrent attempts to stop phenytoin after 10 months of seizure freedom led to relapse of seizures. It was decided to continue phenytoin treatment despite side effects of ataxia and cognitive dysfunction

^†^Likely to be a side effect of phentytoin treatment

#### Patient 1

Patient 1 is a 10-year-old girl who presented with tonic seizures with apneas at the age of 4 months, followed by tonic–clonic seizures. Early development before seizure onset was normal; she currently has moderate psychomotor retardation and ataxia, which could, in part, be due to phenytoin treatment. She was initially treated with valproate and carbamazepine. Later, topiramate was added and valproate was stopped. At 7 months of age, she was diagnosed with a mitochondrial encephalopathy, based on the findings of a muscle biopsy showing decreased adenosine triphosphate production combined with decreased complex II and III activity. Episodes of severe seizures with saturation drops were treated with intravenous phenytoin. As it was noticed that this had an immediate, but also longer-lasting, favorable effect, she was treated with increasing dosages of phenytoin over the following years. Seizure severity and frequency decreased with increasing phenytoin levels (Fig. 1). She showed a remarkable dependency on increasingly high phenytoin levels, with seizures occurring immediately when levels dropped below approximately 20 mg/l initially and 30 mg/l currently (therapeutic range for total phenytoin levels 8–18 mg/l). The parents therefore determined phenytoin levels at least twice weekly, to adjust dosage accordingly. She tolerated high phenytoin levels well. With increasing seizure control, her developmental progress improved. A de novo heterozygous mutation in *SCN8A* was detected in this patient, c.779T>C, p.Phe260Ser, which was indicated to be damaging by in silico prediction. The mitochondrial dysfunction is thought to be secondary to the underlying monogenic epileptic encephalopathy. The clinical phenotype has been described previously [4].

#### Patient 2

Patient 2 is a 2-year-old boy with seizure onset at the age of 5 months. Seizures were characterized by tonic spasms with cyanosis, and moments of staring with apnea at presentation. Psychomotor development slowed after seizure onset and he has, up to now, not developed speech. The boy was initially treated with phenobarbital and valproate. Later, levetiracetam, clobazam, and topiramate were subsequently added. Clobazam was stopped because of behavioral problems. While on this medication, he had a seizure frequency varying from once per 5 weeks to 3 times daily. Frequent episodes of otitis seemed to trigger the seizures. At the age of 14 months, phenytoin was introduced because of increasing seizure frequency. Upon the addition of phenytoin, the seizure frequency decreased. Currently, he has been seizure-free for 6 months on treatment with phenobarbital (currently being phased out), valproate, and phenytoin, with total phenytoin levels between 14 and 24 mg/l, and psychomotor development is improving. By targeted array analysis of epilepsy genes, a de novo heterozygous mutation in *SCN8A*, c.4787C>G (p.Ser1596Cys) was detected. In silico prediction indicated this mutation as possibly damaging.

#### Patient 3

Patient 3 is a 7-year-old boy who had his first seizure at the age of 3.5 months. Seizures were tonic, with apnea, bradycardia, and profound cyanosis, and came in clusters. They were often provoked by infections or fever. In the first 2 years of life, he had 3 episodes of status epilepticus. He was initially treated with valproic acid, after which clobazam and carbamazepine were added. Carbamazepine was later replaced by oxcarbazepine, and valproic acid was stopped. Lamotrigine was tried for 2 months but stopped because he developed a rash. After another cluster of seizures at the age of 8 months, phenytoin was started. With total phenytoin levels between 12 and 20 mg/l, he did not have any further seizures. Four repeated attempts to decrease the phenytoin dose resulted in improvement of cognitive and motor function, but exacerbation of seizure activity, underlining the effectiveness of phenytoin to control seizures in this patient. On occasion, phenytoin levels were high (>30 mg/l) without the boy experiencing complaints of intoxication, although he did have gingival hyperplasia since the start of treatment. At the age of 7 years, brain magnetic resonance imaging showed marked cerebellar atrophy, possibly as a side effect of phenytoin treatment. Again, an attempt was made to decrease the phenytoin dosage and to replace phenytoin with valproic acid. However, at total phenytoin levels <10 mg/l he started to have seizures again and phenytoin treatment was continued. Targeted array analysis of epilepsy genes showed a de novo heterozygous *SCN8A* mutation, c.5610A>T p.Glu1870Asp, which was predicted to be possibly damaging.

#### Patient 4

This patient is an 8-year-old girl with seizure onset at the age of 6 weeks, after which she developed tonic, tonic–clonic, myoclonic, and partial seizures. Multiple electroencephalographic recording showed multifocal epileptiform activity of frontal, central, parietal, and temporal origin. At the age of 9 months treatment consisted of phenytoin, mysoline, and clonazepam. She had an average of 10 tonic–clonic seizures with apnea and cyanosis lasting for 2.0–2.5 min each day. Two episodes of status epilepticus occurred at the age of 2 years, when treated with levetiracetam, phenobarbital, zonisamide, clonazepam, and phenytoin. She was treated with an extra bolus of phenytoin intravenously (IV) and midazolam IV. At the second episode low phenytoin serum concentration (<1.6 mg/l) was detected and she was given additional phenytoin IV. Thereafter, her daily phenytoin dosage was gradually increased from 7.5 mg to 36.0 mg 3 times daily, and a serum concentration of 7.1 mg/l was achieved, after which no major seizures occurred. Total phenytoin concentrations were monitored and fluctuated between 2.8 and 8.4 mg/l. At the age of 8 years, the cosmetic side effects of phenytoin (hypertrichosis) were the reason for withdrawal of phenytoin for an 8-week period, during which the patient immediately developed almost daily tonic and tonic–clonic seizures during sleep. At that time a genetic diagnosis was obtained using whole-exome sequencing, which showed a de novo SCN8A missense mutation c.2952C>G p.Asn984Lys [7]. Phenytoin was reintroduced and the seizures stopped. Recently, the electrophysiological properties of this mutation were studied elsewhere, and the result predicted premature channel opening and hyperactivity of neurons, compatible with the gain-of-function effects reported previously [7].

## Discussion

Here we describe 4 patients who carry a missense mutation in *SCN8A*, in which seizure control is achieved by treatment with (high-dose) phenytoin. Although this series of patients is relatively small, the reoccurrence of seizures when serum phenytoin levels were low in patient 1, and the reoccurrence of seizures after multiple attempts to withdraw phenytoin in patients 3 and 4, provide a direct link between the genetic defect in these patients and their drug response.

Phenytoin is a sodium channel blocker and binds at a receptor site in the pore of sodium channels, decreases the sodium influx, and thereby decreases the excitability of the neuron. Given the fact that phenytoin is one of the few sodium channel blockers targeting only the sodium channel and no other molecular targets in the brain, this might explain the extraordinary response to phenytoin treatment in our patients, in contrast with the response to treatment with other sodium channel blockers. Carbamazepine, another frequently used sodium channel blocker in epilepsy, is thought to have a similar working mechanism to phenytoin. However, the affinity of carbamazepine for inactivated sodium channels is around three times lower than that of phenytoin [10]. Phenytoin works in a frequency-dependent way: it is found primarily to inhibit high-frequency firing but not low-frequency firing, which explains the blocking effect of phenytoin on repetitive discharges as seen in epilepsy, while not interfering with normal neuronal firing. Therefore, phenytoin could be an important treatment option in patients with *SCN8A*-related epilepsy because it is primarily expected to block the increased sodium current through the mutated Nav1.6, but not to affect the function of other VGSC not affected by the *SCN8A* mutation. Side effects of phenytoin include ataxia, nystagmus, cerebellar atrophy, and gingival hyperplasia. Cardiotoxicity (hypotension, prolonged QT interval, arrhythmias) is rare after oral administration but is an important side effect after rapid phenytoin administration IV. The therapeutic range of total phenytoin is a serum level between 10 and 20 mg/l (free phenytoin 1–2 mg/l). Total serum levels >20 mg/l may cause nystagmus, ataxia, and tremor, while total serum levels >40 mg/l can lead to lethargy, confusion, seizures, and coma [11].

The reported patients experienced side effects of phenytoin. However, both the physician and the patients’ parents considered the beneficial effects to outweigh the side effects. In patient 1, only very high serum levels of >30 mg/l led to better seizure control. Neurological side effects were acceptable, which could possibly be explained by the alteration in Nav1.6 function and neuronal excitability, making her probably less sensitive to phenytoin toxicity.

A previous functional study of the *SCN8A* mutation in patient 4 demonstrated a gain-of-function effect in line with the effect observed for the first *SCN8A* patient that was described [7]. However, both gain- and loss-of-function effects of *SCN8A* mutations have been described and further functional studies of *SCN8A* mutations and corresponding phenytoin response must be carefully examined to ensure adverse reactions are avoided [3, 7, 8]. The described loss-of-function effects of 2 different missense mutations may indicate a dominant negative effect leading to a severe phenotype. Alternatively, it remains possible that gain-of-function effects have gone unobserved owing to the nature of the assay and cell system used for functional characterization [3, 7]. Further evaluation of putative loss-of-function mutations is needed, as disease could be aggravated by phenytoin treatment in patients with loss-of-function *SCN8A* mutations. It therefore remains uncertain if a beneficial effect of phenytoin depends on the functional effects of missense mutations. Future studies are also needed to overcome the low number of observations and lack of control of confounders. However, based on the findings in the abovementioned patients we consider treatment with (high-dose) phenytoin as a possible treatment option in patients with difficult-to-control seizures due to an *SCN8A* mutation. Dosing should be titrated gradually and plasma levels above the supposed therapeutic window (i.e., “supratherapeutic”) might be required in order to obtain acceptable seizure control. Owing to saturation pharmacokinetics of (phos)phenytoin, it is important to wait until steady state is reached before increasing the dose further, especially when aiming for supratherapeutic plasma levels. Finally, our observations should be interpreted with care given the low number of patients and lack of control of confounders. Further observations and in vitro or in vivo modeling of the response of SCN8A mutations to sodium blockers are needed.

## Electronic supplementary material

Below is the link to the electronic supplementary material.

ESM 1 (PDF 497 kb)
